# Supplementary material for: Association between oral microbiome and seven types of cancers in East Asian population: a two-sample Mendelian randomization analysis
Source: Front Mol Biosci. 2023 Nov 21;10:1327893. doi: 10.3389/fmolb.2023.1327893 (PMC10702768; doi:10.3389/fmolb.2023.1327893)
Supplement: Supplementary file 1 [file DataSheet1.ZIP › Supplementary Materials/MR plots of saliva/saliva═╝/colorectal cancer/pheno.2599_to_colorectal cancer_forest.pdf]

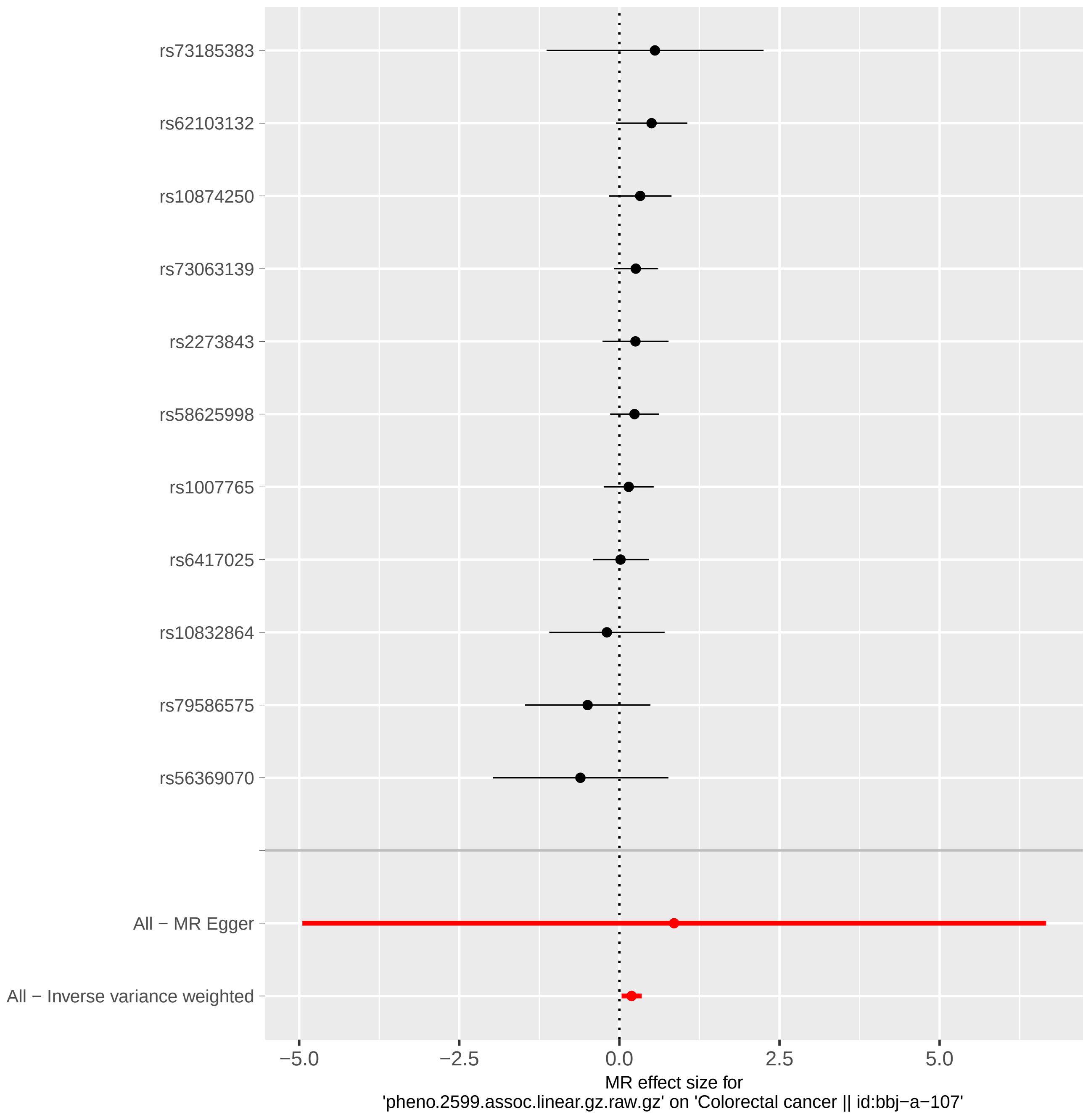
rs73185383
rs62103132
rs10874250
rs73063139
rs2273843
rs58625998
rs1007765
rs6417025
rs10832864
rs79586575
rs56369070
All − MR Egger
All − Inverse variance weighted
−5.0
−2.5
0.0
2.5
5.0
MR effect size for
'pheno.2599.assoc.linear.gz.raw.gz' on 'Colorectal cancer || id:bbj−a−107'
